# Herbivore seasonality responds to conflicting cues: Untangling the effects of host, temperature, and photoperiod

**DOI:** 10.1371/journal.pone.0222227

**Published:** 2019-09-05

**Authors:** Mariana Abarca

**Affiliations:** Department of Biology, Georgetown University, Washington, DC, United States of America; Pennsylvania State University, UNITED STATES

## Abstract

Organisms from temperate ecosystems experience a cyclic alternation of favorable seasons, when they can grow and develop, and unfavorable periods, characterized by low temperatures and reduced resource availability. A common adaptation to these changing conditions is to undergo a state of metabolic arrest triggered by environmental cues (e.g. diapause) during the unfavorable periods. Altered environmental conditions resulting from global change can expose organisms to contradictory cues, potentially triggering maladaptive responses. Here, I compared the performance of an oligophagous butterfly when experiencing consistent vs contradictory environmental cues by manipulating temperature, daylength, and host plant in the laboratory. I implemented a fully factorial design with realistic temperature and photoperiodic regimes to resemble environmental conditions during mid-summer and the summer-autumn transition within the focal species’ range. To assess the role of host plant at mediating the effects of abiotic factors, larvae were fed foliage of either a high or a low-quality host species. Decreasing daylength was the primary cue inducing diapause; however, feeding on a low-quality host at low temperatures also induced diapause in larvae growing under constant summer daylength. Conversely, exposure to high temperatures while feeding on a high-quality host occasionally overruled the diapause-inducing effect of decreasing daylength. Feeding on a high-quality host mitigated the lethal effects of cold, but not of hot temperatures. In addition, exposure to cold temperatures resulted in a significant reduction of pupal mass only under decreasing daylength. These results indicate that responses to environmental stressors in this multivoltine butterfly differ across the growing season according to the eco-physiological state of individuals (whether they undergo direct development or diapause). Traits related to oligophagy, such as sensitivity to multiple cues for diapause induction, as well as some of its consequences, such as the occurrence of overlapping generations, are likely to mitigate some of the detrimental effects of global change.

## Introduction

Temperate ecosystems are characterized by the alternation of favorable and unfavorable periods for growth and reproduction. Plants and animals native to these environments have evolved physiological mechanisms that allow them to cope with seasonal change by minimizing their metabolic activities during unfavorable periods [1,2]. Environmental conditions such as daylength, temperature, and food quality trigger physiological responses that result in synchronization of life-history activities with seasonal change [1,3,4]. There is increasing concern that the novel environmental conditions produced by global change, including altered temperature regimes and novel hosts, can expose organisms to contradictory stimuli, triggering maladaptive responses and/or phenological mismatches [5–7].

A key adaptation of numerous temperate insects is to undergo diapause during the unfavorable season [1]. Diapause is a state of metabolic arrest triggered by token stimuli (e.g. short or decreasing daylength). These stimuli occur before the onset of harsh environmental conditions and trigger physiological processes that prepare organisms to withstand low temperatures and prolonged periods of food deprivation [1,8]. In multivoltine populations diapause is a plastic trait; each individual has the potential to either complete development and start a new generation (direct development) or enter diapause and overwinter until the following year. These alternative phenotypes allow for the occurrence of one, two or several generations per year, depending on local growing season length [9,10]. The probability that a given multivoltine individual will initiate direct development vs diapause depends on environmental conditions [1]. The most common cue triggering diapause induction is short or decreasing daylength [1,8]; however, low temperature, reduced moisture and low or decreasing host quality can also trigger diapause induction [1,3,11,12]. Individuals that overwinter face unique challenges, as they are exposed to cold temperatures and cannot acquire resources for prolonged periods. Diapause induction typically involves the activation of pathways that alter development and growth rates [13] and increase the accumulation of metabolic reserves, including lipids and carbohydrates [14]. Thus individuals that enter diapause are typically larger than those that undergo direct development [15].

Insect development time and adult size also vary in response to temperature. Warmer temperatures (within physiological limits) accelerate growth rate and result in reductions of both development time and adult size [16,17]. Thus, insects growing at low temperatures tend to be larger and more fecund than their conspecifics growing at high temperatures [16–18]. Likewise, variation in foliage quality within and among host species can result in significant differences in herbivore adult size and development time [19,20]. For example, fall webworms (*Hypantria cunea*) feeding on *Prunus serotina* took about 18% longer to complete larval development and attained half the size of their conspecifics feeding on red mulberry (*Morus rubra*) [19]. In general, larger individuals have increased reproductive potential [21] and are more likely to survive the overwintering period [15,22,23]. Therefore, both low-quality foliage and thermal stress can significantly reduce herbivore fitness.

Daylength, temperature, and foliage quality [24,25] drop at the end of the growing season. Thus, these environmental conditions can act as seasonal cues that usually reinforce each other. However, under global change conditions, photoperiodic change remains unaffected while temperature regimes [26] as well as host suitability change. Alterations in host identity [27], availability, and quality [28] result from multiple phenomena such as range shifts [29], phenological mismatches [30–32] and the direct effects of increased temperatures on foliage chemistry [28]. These altered environmental conditions can affect diapause induction as well as resource intake and assimilation by herbivorous insects.

Many insect populations are in decline as they face a variety of stressors, including novel climates, habitat destruction, declining host plant quality, and altered plant communities [27,33–36]. Therefore, understanding herbivore responses to different combinations of environmental stressors, such as increased temperatures and novel hosts in a seasonal context, is necessary to inform population forecasts and conservation efforts. This study presents a laboratory experiment manipulating temperature, photoperiod and host quality to identify their combined effects on life history traits and phenological responses of the silver-spotted skipper (*Epargyreus clarus*), a multivoltine, oligophagous butterfly native to North America that has incorporated invasive species into its diet [37,38]. Experimental regimes with diel oscillation in temperature as well as realistic variation in daylength were implemented to i) identify the environmental cues triggering diapause induction, ii) evaluate the role of host quality in mediating thermal stress, and iii) assess the effects of warmer-than-average temperature regimes on fitness correlates (survival, development time and pupal mass). The combinations of photoperiod and temperature implemented here allowed for a comparison of the effects of warming in the middle and at the end of the growing season. I expected high temperatures to result in increased mortality, reduced pupal mass, and, when occurring at the end of the season, to override the diapause-inducing effect of decreasing daylength. I also expected high host quality to mitigate thermal stress, as seen in other lepidopterans [18].

## Materials and methods

### Study species

Silver-spotted skippers have a broad distribution, including southern Canada and the continental USA, where they have two to three generations per year and overwinter as pupae. Larvae feed on a variety of leguminous hosts, and they build characteristic leaf shelters [39]. Host species typically used by *E*. *clarus*, differ in quality [38], with *Pueraria montana* (Lour.) having higher nitrogen (~ 4%) and water (~75%) content by mass, than *Wisteria sinensis*, Sims. (nitrogen: ~2.5%, water: ~60%). Furthermore, larvae feeding on *P*. *montana* have increased survival, shorter development time, and larger pupae compared to those feeding on *W*. *sinensis* [38]. These two invasive species occupy opposite ends in the host-quality gradient used by wild *E*. *clarus* [38]. In addition, these species coexist in space and time, their foliage is available throughout the *E*. *clarus* activity period, and they produce new leaves throughout the growing season. Therefore, they were selected as the high- and low-quality hosts.

### Experimental design

**Fig 1:**
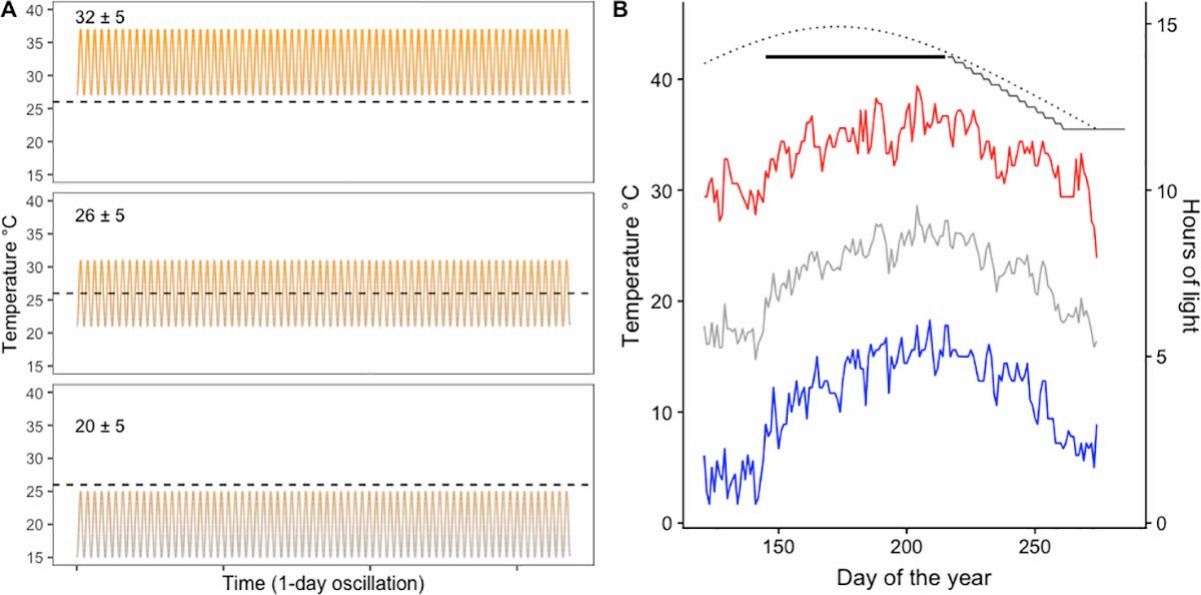
Experimental conditions. A) Experimental temperature regimes; dashed horizontal lines denote 26°C to facilitate visual comparison. B) Daylength (dotted black line, left axis) and temperature (right axis) during the growing season in the study area. Lines show average daily minimum (blue), maximum (red) and mean (grey) temperatures from May to September from 2000 to 2016 as recorded by a weather station in Aberdeen Phillips Field MD, US, (elevation:14 m, latitude: 39.467, longitude: -76.117). Bold lines denote experimental photoperiodic regimes: constant (black) and decreasing (grey).

To assess the effects of host plant, photoperiod and temperature on silver-spotted skipper seasonality, I implemented a full factorial design, exposing individuals in growth chambers (Models 130 VL and 136VL, Percival Scientific, Perry, IA, USA) feeding on either a high (*P*. *montana*) or a low-quality host (*W*. *sinensis*) to each of three oscillating, sinusoidal diel temperature regimes (mean: 20, 26, 32°C; 5°C amplitude, Fig 1A) and at either a constant (14 hrs. of light) or decreasing photoperiod; in which daylength decreased from an initial 14 hrs. of light per day to 12 hrs. 50 min. over seven weeks (Fig 1B). Daylength decreased in regular 10 min steps that occurred alternately after 3 or 4 days, Fig 1). These temperature conditions are similar to those typically experienced in Maryland, USA (39° N, 77° W), where the mean air temperature is close to 26°C during mid-summer and close to 20°C at the end of the growing season. Mean daily temperatures of 32°C (the hottest treatment) are not common, but they occur during heatwaves in this area (Fig 1).

To ensure uniform exposure of larvae to photoperiodic conditions, eggs of *E*. *clarus* (1 to 3 days old) were assigned to each of the temperature treatments under a constant 14 hrs. of light photoperiod, and synchronously hatching caterpillars were transferred to each of the twelve treatments upon hatching (N = 40 caterpillars per treatment). Caterpillars were kept under their corresponding treatments until adult emergence or diapause induction. I used one growth chamber for each daylength-temperature combination, so larvae feeding on both hosts hatched synchronously and were kept in in individual deli containers inside the same chamber. I recorded development time and developmental stage of each individual five days per week, making sure the period between inspections was no longer than 48 hrs. Freshly-cut foliage of the corresponding host was replenished during each inspection. When completion of larval development took longer than 7 weeks, the minimum daylength was maintained constant thereafter. Individuals were assumed to have entered diapause when they failed to emerge after a reasonable period. This period varied in length according to treatment temperature; 41 days for individuals kept at 20 + 5°C, 26 days for those kept at 26 + 5°C and 20 days for pupae exposed to 32 + 5°C. For comparison, pupae from a different experiment kept at constant 26°C emerged after 13 days [40]. Pupae presumed to be in diapause were kept in the laboratory under overwintering regimes; 95% of overwintering pupae survived to emergence the following April (~8 months later). Pupae were sexed and weighed within three days of pupation using a digital balance (Mettler Toledo MX5). In addition to development time, I recorded the incidence of intercalary larval instars (Table 1), which indicates stress, as deviations from the typical instar number (five in the case of *E*. *clarus*) can occur under suboptimal conditions (Esperk et al., 2007).

**Table 1:** Development time (days) of larvae (hatch to pupation) and non-diapausing pupae (pupation to emergence), and incidence of intercalary instars under each temperature and photoperiod combination. All treatments started with 40 larvae.

**Constant photoperiod**				
**Mean temperature**	**Host** **quality**	**Larva** **mean ± SE (N)**	**Pupa** **mean ± SE (N)**	**Incidence of intercalary instars (%)**
**20**	**High**	**49 ± 2 (29)**	**19 ± 1 (19)**	**3**
	**Low**	**69 ± 2 (9)**	**22 ± 1 (4)**	**78**
**26**	**High**	**25 ± 1 (39)**	**13 ± 0.1 (39)**	**8**
	**Low**	**38 ± 1 (36)**	**12 ± 0 (35)**	**58**
**32**	**High**	**21 ± 0 (21)**	**11 ± 0 (21)**	**33**
	**Low**	**35 ± 1 (20)**	**10 ± 0 (20)**	**100**
**Decreasing photoperiod**				
**20**	**High**	**44 ± 1 (25)**	**-**	**0**
	**Low**	**62 ± 2 (10)**	**-**	**50**
**26**	**High**	**28 ± 0 (40)**	**-**	**3**
	**Low**	**39 ± 1 (32)**	**-**	**73**
**32**	**High**	**27 ± 1 (28)**	**11.00 ± 0 (5)**	**39**
	**Low**	**41 ± 1 (25)**	**-**	**84**

All individuals included in this experiment were part of a laboratory colony of *E*. *clarus* founded in summer 2016 with wild-caught individuals from the Washington, DC (38.89, -77.04) and Maryland (39.027, -76.79) area, USA. This colony was also supplemented with wild-caught individuals in early 2017, the year when experiments were carried out. Care was taken to ensure an even mix of eggs from multiple females was included in each treatment.

Finally, to estimate voltinism in the study area (39 N) I calculated generation time of *E*. *clarus* feeding on wisteria and kudzu at 26 + 5°C of diapausing (egg + larva) and non-diapausing (egg + larva + pupa) individuals. I assumed development time of eggs was 6 days as reported for this species at constant 26°C [40].

### Statistical analyses

To assess the effect of treatment on each response variable, I fit linear (pupal mass) and generalized linear (development time, survival to pupation, and diapause induction) models, with temperature (3 levels), host, sex, daylength and their interactions as independent variables. While many lepidopterans exhibit marked sexual dimorphism, *E*. *clarus* does not. Hence, sex was not a significant factor in the initial models and was thus removed. Development time data could not be analyzed using a linear model because the distribution of residuals deviated from normality, thus, I implemented a generalized linear model with a Gamma distribution and inverse link function including photoperiod, temperature host and their interactions as independent variables. Models evaluating diapause induction and survival assumed a binomial distribution with a logit link function. The model analyzing survival assessed the probability of larval survival to pupation given the rearing conditions, while the model analyzing diapause induction assessed the probability of these survivors to enter diapause. A type II deviance test implemented in the Anova function of the car package in R was used to evaluate the significance of the predictors of all generalized linear models [41]. Non-significant interaction terms were removed from models. Residuals were visually inspected for model validation. Where significant terms were present, least-squares means post-hoc tests with Bonferroni adjustment were conducted using emmeans R package [42].

## Results

**Fig 2:**
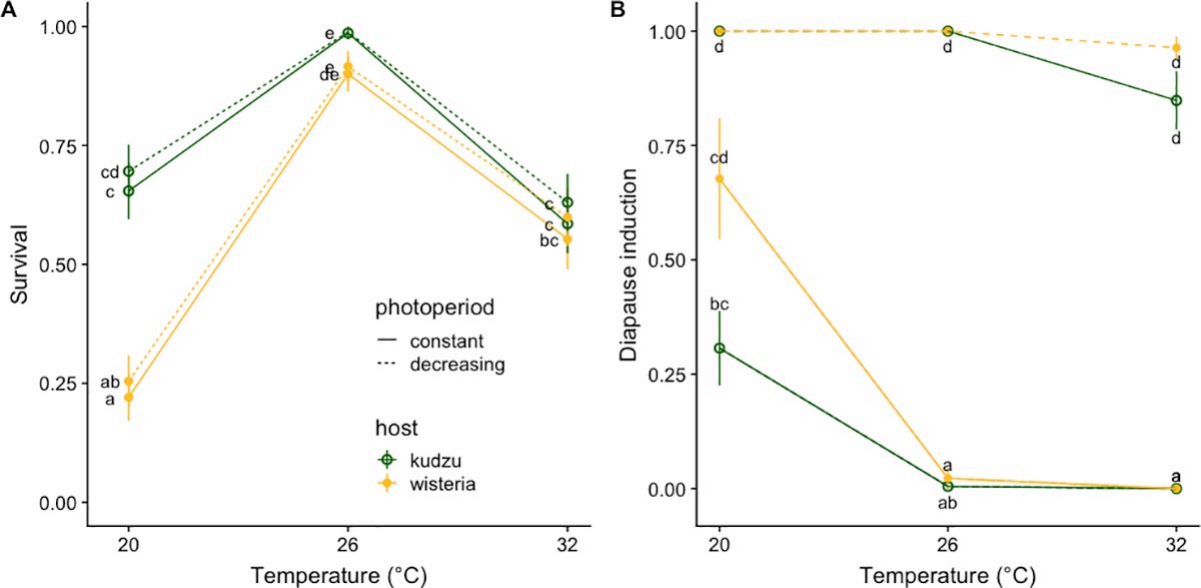
Survival and diapause induction. A) Probability of survival and B) diapause induction (LS means ± SE) of individuals kept at each of three oscillating temperature regimes (mean ± 5), feeding on either a high quality (green, open circles, kudzu, *Pueraria montana*) or a low quality (yellow, solid circles, wisteria, *Wisteria sinensis*) host. Solid lines correspond to constant and dashed lines to decreasing daylength. Letters designate significantly different groups according to post hoc least squares tests with Bonferroni correction.

Survival patterns (Fig 2A) and incidence of intercalary instars (Table 1) indicate that experimental treatments with mean temperatures of 20°C and 32°C were stressful for *E*. *clarus*. Temperature (*χ*_2_^2^ = 111.839, *P* < 0.001), host (*χ*_1_^2^ = 22.728, *P* < 0.001), and their interaction (*χ*_2_^2^ = 15.097, *P* < 0.001, Fig 2A) but not photoperiod (*χ*_1_^2^ = 0.685, *P* = 0.41) affected caterpillar survival. At 26± 5°C survival was greater than 80% regardless of host. Under thermal stress, feeding on a high-quality host resulted in increased survival at low (20°C), but not at high temperatures (32°C, Fig 2A). For the survivors, temperature (*χ*_2_^2^ = 58.83, *P* < 0.0001), daylength (*χ*_1_^2^ = 336.27, *P* < 0.0001), and host (*χ*_1_^2^ = 6.52, *P* = 0.011) explained 80% percent of the variation in diapause induction. Daylength was the strongest cue, as 78% of individuals reared under a decreasing daylength entered diapause regardless of host and temperature. Only five individuals in this treatment did not enter diapause, and all of them were feeding on the high-quality host at 32°C (Fig 2B). When experiencing constant, long days only 12% of individuals entered diapause and most of them were reared at 20°C. *Epargyreus clarus* individuals from a separate assay growing at constant temperatures and daylength, rather than the more realistic fluctuating conditions examined here, exhibited similar patterns of survival and diapause induction (S1 Appendix).

**Fig 3:**
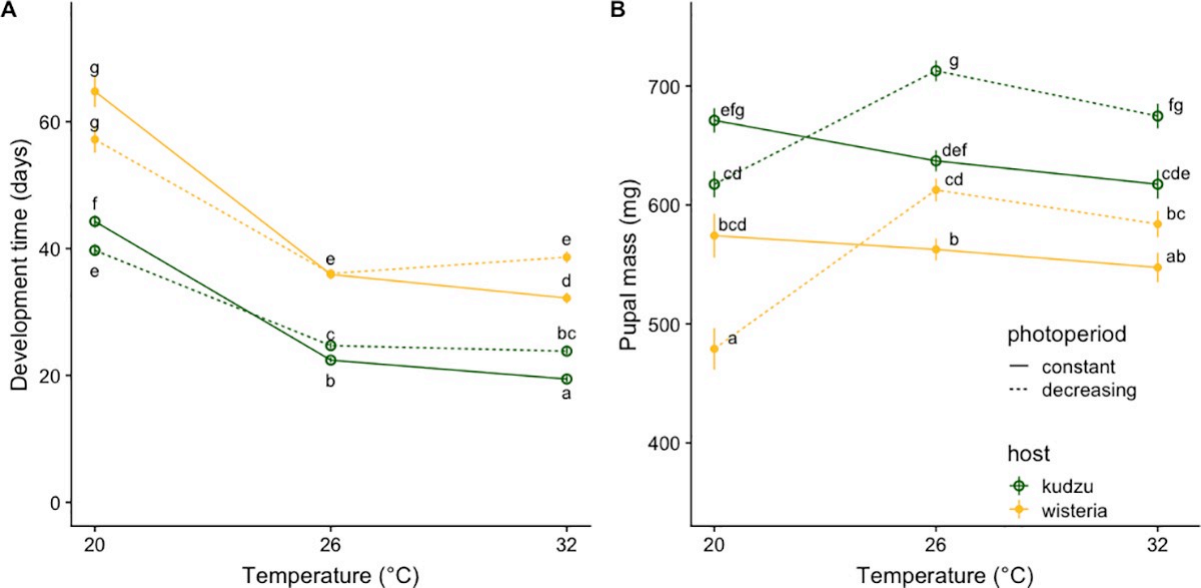
Development time and pupal mass. **A)** Development time (instar 1 to prepupae; LS means ± SE) and **B)** pupal mass (mean ± SE) of individuals kept at each of three oscillating temperature regimes (mean ± 5), feeding on either a high quality (green, open circles, kudzu, *Pueraria montana*) or a low quality (yellow, solid circles, wisteria, *Wisteria sinensis*) host. Solid lines correspond to constant and dashed lines to decreasing daylength. Letters designate significantly different groups according to post hoc least squares tests with Bonferroni correction.

Median development time (egg to pupa) varied from 20 to > 60 days depending on host plant and temperature (Fig 3A). While feeding on a high-quality host consistently shortened development time (*χ*_1_^2^ = 955.45, *P* < 0.0001, Fig 3A), the effect of temperature (*χ*_2_^2^ = 1412.34, *P* < 0.0001) was mediated by daylength (daylength:*χ*_1_^2^ = 4.51, *P* = 0.034, daylength -temperature-host interaction: *χ*_2_^2^ = 8.70, *P =* 0.013). At 20°C, larvae growing under a decreasing daylength and feeding on the high-quality host accelerated their development (10% reduction in development time; Fig 3A). By contrast, at 32+ 5°C, exposure to a decreasing daylength significantly lengthened development time. Individuals feeding on the low-quality host experienced a development time extension of 17% and those feeding on the high-quality host of 20% (Fig 3A). Under typical summer temperatures (26 + 5°C), development time of larvae feeding on the low-quality host did not significantly differ between daylength treatments; however, those feeding on kudzu experienced a 12% increase in development time when exposed to decreasing daylength (Fig 3A).

Temperature, daylength, host, and their interactions explained 51% of the variation observed in pupal mass (*F*_7,307_ = 45.4, *P* < 0.0001, Table 2, Fig 3B), with individuals feeding on the high-quality host yielding significantly larger pupae than those feeding on the low-quality host. The effect of daylength on pupal mass was temperature-dependent, as decreasing daylength resulted in smaller pupae at low temperatures (8% kudzu, 16% wisteria) and larger pupae at high temperatures (~ 10% kudzu, ~ 8% wisteria); this effect was consistent between hosts (Fig 3B).

**Table 2:** Anova table (type II) of the effect of temperature (3 levels), host, daylength and their interactions on pupal mass of *E*. *clarus*.

Source of variation	df	Sum of squares	*F*	*P*
temperature	2	78906	12.75	< 0.0001
host	1	618222	201.89	< 0.0001
photoperiod	1	66309	21.65	< 0.0001
temperature × host	2	13391	2.19	0.11
temperature × photoperiod	2	223839	36.55	< 0.0001
host × photoperiod	1	13611	4.44	0.04
temperature × host × photoperiod	2	966	0.16	0.85
residuals	303	927854		

**Fig 4:**
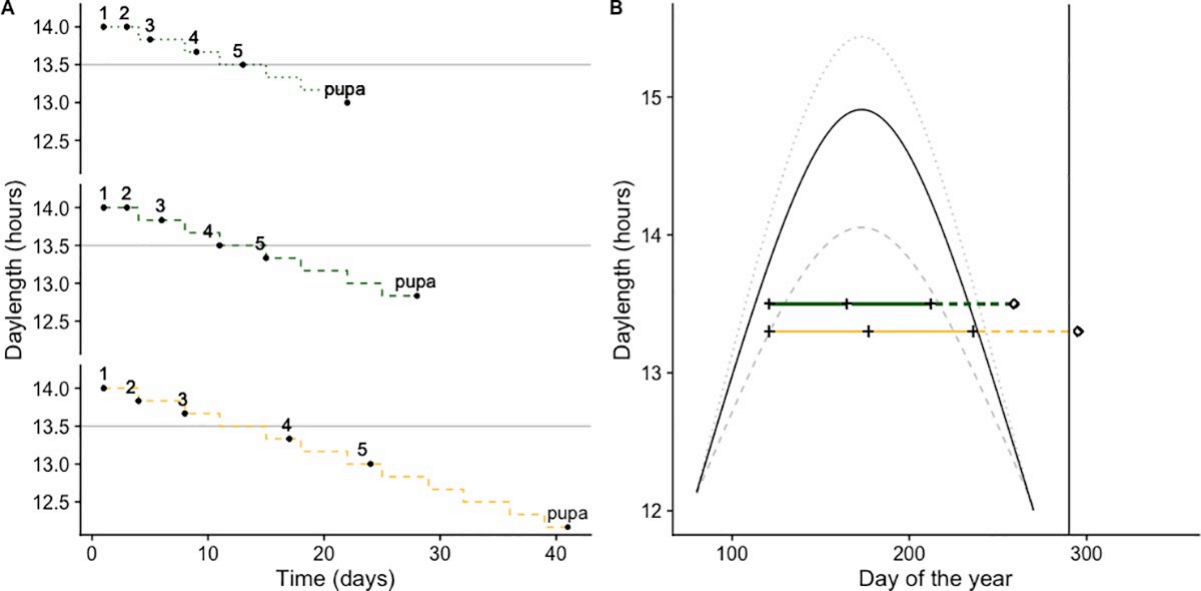
Daylength. A) Hours of light experienced by each larval instar of *E*. *clarus* feeding on kudzu (green) and wisteria (yellow) growing at 32 + 5°C and decreasing daylength. Dashed lines correspond to individuals that entered diapause and dotted line to the 5 individuals that did not enter diapause. Black points indicate mean time to each of the five typical larval instars and to pupation (intercalary molts are not shown to improve clarity; development time of intercalary instar “3.5” is considered part of instar 3). Similar plots showing larvae at 26 + 5°C and 20 + 5°C are available as supplementary information. B) Natural change in daylength at three latitudes encompassing *E*. *clarus* range, 43.6 N (southern Canada, dotted line), 39 N (study area, Maryland, USA, solid line) and 29.7 N (southern USA, dashed line), values were obtained using the R package geosphere [43]. Horizontal solid lines (green: kudzu; yellow: wisteria) represent generation time (egg to adult emergence, crosses indicate adult emergence) of *E*. *clarus* assuming May 1st as the time of first flight and an ambient temperature of 26 + 5°C. Dashed lines represent development time (egg to pupation, pupation indicated by diamonds) of individuals undergoing diapause. Vertical black line indicates October 17th, when early frosts have been recorded in the study area [40].

For larvae experiencing decreasing daylength, differences in development time induced by temperature and host resulted in differential exposure to light among larval instars (Fig 4A). For example, first instar larvae feeding on the high-quality host at 32 + 5°C molted to 2^nd^ instar in less than 3 days. Thus, they experienced a constant daylength of 14 hours (Fig 4A). In all other cases, each larval instar experienced at least one daylength decrement (Fig 4A, S2 Appendix). The five individuals that did not enter diapause under decreasing daylength (dotted line, Fig 4A) experienced light during more than 13.5 hours per day from instars 1 to 4. By contrast, larvae in all other treatments experienced daylengths equal or shorter than 13.5 hours by the time they molted to 4^th^ instar. Differences in instar-specific daylength exposure are likely to occur within populations due to differential host consumption (Fig 4B). Assuming May 1^st^ as the time of first flight, *E*. *clarus* in the DC-Maryland area (39 N) feeding on both kudzu and wisteria would likely have three generations (two with direct development and one that overwinters). However, the risk of developing interrupted generations would be larger for those feeding on wisteria, as pupation time is predicted to occur in mid- October, when frost risk increases (Fig 4B).

## Discussion

Daylength was the main cue inducing diapause in *E*. *clarus* and it mediated the effects of environmental stress on larval performance. These results indicate that the effects of stressful temperature regimes and low- quality foliage differ across the growing season. Individuals exposed to decreasing daylengths were not only more likely to express diapause, they were also more sensitive to low temperatures. While both low (20 + 5°C) and high (32 + 5°C) temperature treatments increased mortality regardless of daylength, their sublethal effects on pupal mass differed according to light exposure. Low temperatures at the end of the season (decreasing daylength) resulted in pupal mass reductions of up to 17% when compared to individuals growing at the same temperature but during the beginning of the season (constant daylength). By contrast, larvae exposed to mid (26 + 5°C) and high (32 + 5°C) temperatures at the end of the season produced the largest pupae. Because pupal mass is an important predictor of reproductive outcome and overwinter survival, temperature conditions at the end of the season are likely to influence population size the following year.

While decreasing daylength was the primary cue for diapause induction, both host quality and temperature were able to override its effects, especially in combinations that potentiated their impact on development time (both low or both high). Low host quality and low temperatures lengthened development time and resulted in diapause induction in about 70% of individuals under mid-summer daylength conditions. Conversely, the combination of high host quality and high temperature (which reduced development time) induced direct development even under decreasing daylength in a small fraction of individuals. This flexibility in diapause induction is probably an adaptation that allows *E*. *clarus* to exploit multiple hosts within a broad latitudinal range.

Silver-spotted skippers commonly use hosts of variable quality that coexist in time and space [38]. A single *E*. *clarus* female may lay eggs in multiple hosts over about two weeks (M. Weiss Pers. Comm.; data from an outdoor insectary), which is likely to result in the co-occurrence of a variety of life stages and overlap of generations within populations (Fig 4B). In addition, *E*. *clarus* inhabits a broad latitudinal gradient, encompassing different growing season lengths and photoperiodic conditions (Fig 4B). Under these circumstances, solely relying on a specific critical daylength to induce diapause would be maladaptive. Future studies are necessary to describe gene flow patterns among *E*. *clarus* populations from different latitudes and to determine whether disjunct populations differ in their relative sensitivity to temperature and host quality as diapause-inducing cues.

Photoperiodic induction of diapause has been reported to involve a sequence of processes including light sensitivity, the measurement of hours of darkness (or light) per day, and their accumulation over time (Saunders 1981). Empirical studies have shown that temperature can affect diapause induction during each of these phases [44]. Similarly, host quality may affect diapause induction through its effect on development rate. Relatively fast development and/or growth rates have been linked to the onset of metamorphosis in lepidopterans [3,11], mosquitoes [45] and amphibians [46], while low development rates are associated with developmental arrest.

In this study, only five out of 160 individuals that survived under decreasing daylength did not enter diapause. These five individuals fed on the high-quality host at high temperatures and they developed faster than the rest of their cohort under the same temperature conditions (Fig 4A). As a result of their fast development, these individuals experienced slightly different daylight conditions than the rest of their cohort feeding on the same host; only these individuals were exposed to more than 13.5 hours of light per day during the first 4 larval instars. It is possible that accelerated development during the early instars prevented these individuals from experiencing a “critical daylength” that would trigger diapause induction (e.g. fewer than 13.5 hours of light per day). However, future studies with larger sample sizes would be necessary to corroborate this pattern and to determine whether diapause induction in this species occurs in response to a specific “critical photoperiod” or to falling daylengths over a given time period.

According to the lost generation hypothesis [5], accelerated growth rates occurring in response to warmer temperatures can trigger a mismatch between the seasonal cue inducing diapause (e.g. critical photoperiod) and the ontogenetic stage that is sensitive to it (e.g. early-instar larvae). This mismatch results in the development of an interrupted generation, which starts so late in the season that it is not able to reach the overwintering stage before the first frost and is thus killed. The occurrence of interrupted generations is probably common in silver-spotted skippers as larvae have been observed in the field as late as October in the study area (M. Weiss; Pers., Comm.). It is unclear whether longer and warmer growing seasons would increase or decrease the occurrence of interrupted generations in *E*. *clarus*. However, the co-occurrence of multiple hosts of different quality would likely result in sufficient generational overlap to prevent population collapse even if some individuals fail to enter diapause before the onset of winter. A more immediate threat in this system would be exposure to stressful temperatures (both hot and cold) that can occur as a result of longer growing seasons and irregular weather patterns.

Exposure to temperatures unusually high for the study area (32 + 5°C) induced mortality, but had no detectable effect on the pupal mass and development time of the survivors, indicating some resilience of *E*. *clarus* to heat stress. However, there are other negative effects of heat stress such as decreased longevity, immune function or fecundity, which have all been shown to occur in insects and cannot be ruled out [47–49]. High host quality mitigated the lethal effects of thermal stress at low, but not at high temperatures, regardless of daylength. By contrast, both host plant and daylength mediated the sublethal effects (extended development time, reduced pupal mass) of thermal stress. Under mid-summer daylengths individuals growing on the high-quality host produced larger pupae than those feeding on the low-quality host and there were no detectable differences among temperature treatments (within hosts). By contrast, decreasing daylenth resulted in a violation of the size-temperature rule (larger at cold temperatures), with larvae growing under low temperatures yielding much smaller pupa than those growing under warm (26 + 5°C) and hot (32 + 5°C) regimes. It is unclear whether this pattern of phenotypic plasticity is a byproduct of developmental tradeoffs or an adaptive response. The small size of individuals feeding on the low-quality host could be a direct result of stress, as pointed out by Diamond and Kingsolver (2010). However, for individuals growing under both decreasing photoperiod and low temperature, breaking the temperature-size rule could be adaptive. Pupating at a small size allows for shorter development times, which in turn allows an escape from imminent freezing conditions. In *E*. *clarus*, I found no evidence of higher mortality of these smaller pupae, as 95% of diapausing individuals survived overwintering; however, it is important to note that overwintering conditions in the lab were potentially less stressful than ambient conditions.

Intraspecific changes in host quality can be a reliable indicator of seasonality because foliage composition changes in a predictable way across the season; water and nitrogen concentration are maximized in young foliage, while old leaves are usually tough and rich in phenolic compounds [24,25]. However, global change can decrease the reliability of this cue; for example, warmer temperatures can result in reduced host quality [28] and altered plant community composition may prompt insect host shifts [50], either of which could trigger atypical phenological patterns. In the case of monarch butterflies, exposure to a tropical, evergreen, non-native milkweed may result in the interruption of reproductive diapause [6,51]. In the *E*. *clarus* system, adaptations to oligophagy, such as sensitivity to multiple cues for diapause induction are likely to act as pre-adaptations to global climate change. However, the resilience provided by generational overlap depends on the coexistence of multiple host species. Thus, it is important to maintain diverse plant communities to buffer the detrimental effects of altered temperature regimes on oligophagous insects.

## Supporting information

S1 Appendix Survival and diapause induction at constant temperature. Survival (a) and diapause induction (b) of individuals kept at constant temperatures (20, 26 or 32°C) and constant photoperiod (14hrs light). Initial sample size was 40 individuals per treatment. For detailed rearing conditions see Abarca, M., Larsen, E. Lill, J. Weiss, M. Lind, E. & Ries, L. 2018. Inclusion of host quality data improves predictions of herbivore phenology. *Entomologia Experimentalis et Applicata*. DOI: 10.1111/eea.12715.
(DOCX)

S2 Appendix Light exposure by larval instar. Hours of light experienced by each larval instar of *E*. *clarus* feeding on kudzu (green) and wisteria (yellow) growing at A) 20 + 5°C and B) 26 + 5°C under decreasing daylength. Dashed lines correspond to individuals that entered diapause; black points indicate mean time to each of the five typical larval instars and to pupation (intercalary molts are not shown to improve clarity; development time of intercalary instar “3.5” is considered part of instar 3).
(DOCX)

S1 File Metadata. (DOCX)

S2 File Data. Development time and survival of *E*. *clarus* under experimental conditions.
(CSV)
