# Supplementary material for: Herbivore seasonality responds to conflicting cues: Untangling the effects of host, temperature, and photoperiod
Source: PLoS One. 2019 Sep 5;14(9):e0222227. doi: 10.1371/journal.pone.0222227 (PMC6728043; doi:10.1371/journal.pone.0222227)
Supplement: S1 Appendix — Survival (a) and diapause induction (b) of individuals kept at constant temperatures (20, 26 or 32°C) and constant photoperiod (14hrs light). Initial sample size was 40 individuals per treatment. For detailed rearing conditions see Abarca, M., Larsen, E. Lill, J. Weiss, M. Lind, E. & Ries, L. 2018. Inclusion of host quality data improves predictions of herbivore phenology. Entomologia Experimentalis et Applicata. DOI: 10.1111/eea.12715. (DOCX) [file pone.0222227.s001.docx]

Supporting information: Appendix S1. Abarca, M. Herbivore seasonality responds to conflicting cues: Untangling the effects of host, temperature, and photoperiod.

Figure S1. Survival (a) and diapause induction (b) of individuals kept at constant temperatures (20, 26 or 32°C) and constant photoperiod (14hrs light). Initial sample size was 40 individuals per treatment, see Abarca et al 2018 for a detail description of rearing conditions.


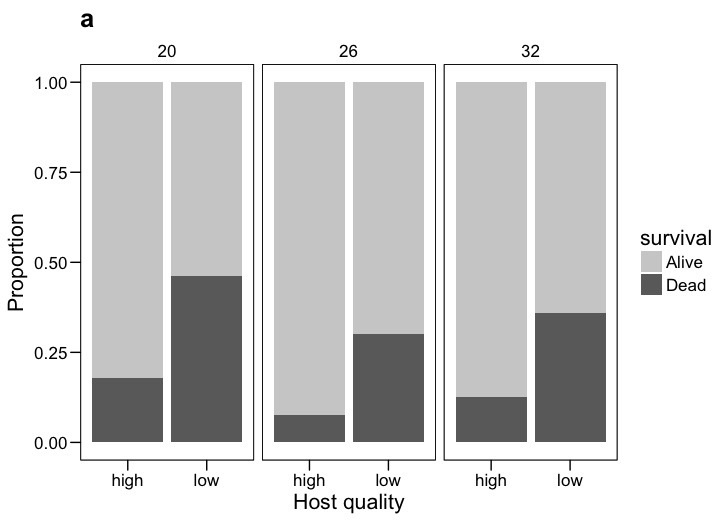


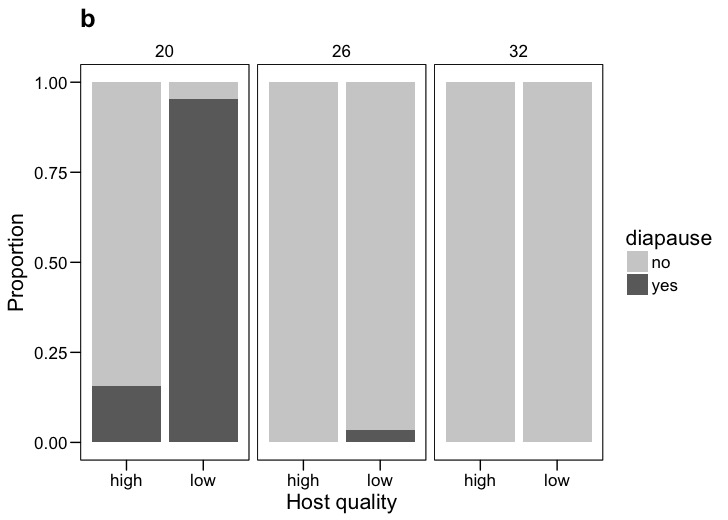


Abarca, M., Larsen, E. Lill, J. Weiss, M. Lind, E. & Ries, L. 2018. Inclusion of host quality data improves predictions of herbivore phenology. *Entomologia Experimentalis et Applicata*. DOI: 10.1111/eea.12715.
