# Supplementary material for: Herbivore seasonality responds to conflicting cues: Untangling the effects of host, temperature, and photoperiod
Source: PLoS One. 2019 Sep 5;14(9):e0222227. doi: 10.1371/journal.pone.0222227 (PMC6728043; doi:10.1371/journal.pone.0222227)

Supporting information: Appendix S2. Abarca, M. Herbivore seasonality responds to conflicting cues: Untangling the effects of host, temperature, and photoperiod.

**Figure S2.** Hours of light experienced by each larval instar of *E. clarus* feeding on kudzu (green) and wisteria (yellow) growing at A) 20 + 5 °C and B) 26 + 5 °C under decreasing daylength. Dashed lines correspond to individuals that entered diapause; black points indicate mean time to each of the five typical larval instars and to pupation (intercalary molts are not shown to improve clarity; development time of intercalary instar “3.5” is considered part of instar 3).


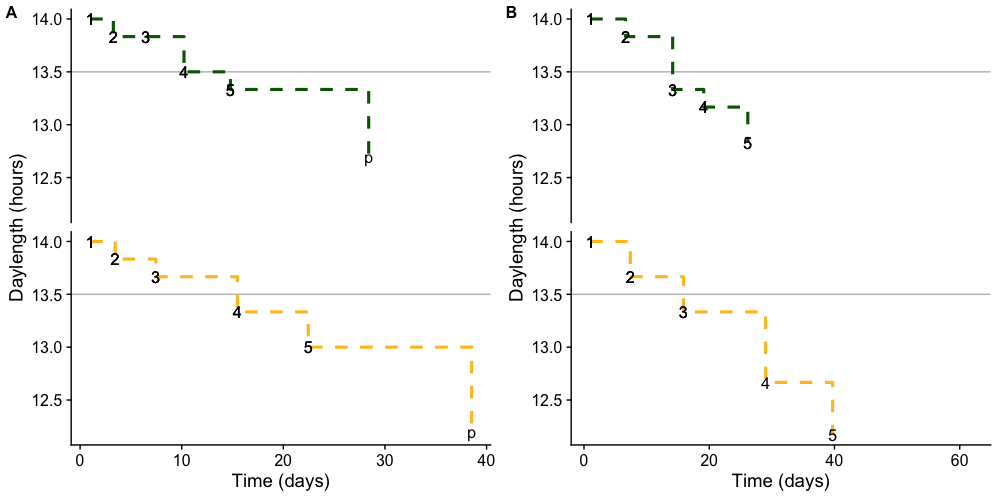

Supplement: S2 Appendix — Hours of light experienced by each larval instar of E. clarus feeding on kudzu (green) and wisteria (yellow) growing at A) 20 + 5°C and B) 26 + 5°C under decreasing daylength. Dashed lines correspond to individuals that entered diapause; black points indicate mean time to each of the five typical larval instars and to pupation (intercalary molts are not shown to improve clarity; development time of intercalary instar “3.5” is considered part of instar 3). (DOCX) [file pone.0222227.s002.docx]
