# Supplementary material for: Herbivore seasonality responds to conflicting cues: Untangling the effects of host, temperature, and photoperiod
Source: PLoS One. 2019 Sep 5;14(9):e0222227. doi: 10.1371/journal.pone.0222227 (PMC6728043; doi:10.1371/journal.pone.0222227)
Supplement: S1 File — (DOCX) [file pone.0222227.s003.docx]

Data from Herbivore seasonality responds to conflicting cues: Untangling the effects of host quality, temperature, and photoperiod.

Fields Description

| id | Individual ID |
| --- | --- |
| temp | temperature regime |
| photo | photoperiod (c: constant, d: decreasing) |
| host | host plant (k: *Pueraria montana*, w: *Wisteria sinensis*) |
| sex | male (m) or female (f) |
| mass | fresh pupal mass (mg) |
| died | Stage at which each individual died  I1 to I6: larval instars  pre: prepupae  pup: pupa  adult: adult |
| survival | From hatching to adult emergence or diapause induction |
| malformations | yes, no |
| diapause | yes: induced diapause, no: direct development |
| instar1 | duration of each lifestage (days) |
| instar2 | duration of each lifestage (days) |
| instar3 | duration of each lifestage (days) |
| instar3.5 | duration of each lifestage (days) |
| instar4 | duration of each lifestage (days) |
| instar4.5 | duration of each lifestage (days) |
| instar5pre | duration of each lifestage (days) |
| instar5pup | duration of each lifestage (days) |
| larvapre | hatch to prepupae (feeding stage, days) |
| larvapup | hatch to pupae (days) |
| pupadt | pupation to adult emergence (non-diapausing individuals) |
| surlarva | survival from hatching to prepupae |
| ninstar | number of larval instars |
